# Microfabrication with Very Low-Average Power of Green Light to Produce PDMS Microchips

**DOI:** 10.3390/polym13040607

**Published:** 2021-02-18

**Authors:** Lucero M. Hernandez-Cedillo, Francisco G. Vázquez-Cuevas, Rafael Quintero-Torres, Jose L. Aragón, Miguel Angel Ocampo Mortera, Cesar L. Ordóñez-Romero, Jorge L. Domínguez-Juárez

**Affiliations:** 1 Centro de Física Aplicada y Tecnología Avanzada, Universidad Nacional Autónoma de México, Juriquilla, Querétaro 76230, Mexico; luc@comunidad.unam.mx (L.M.H.-C.); rquintero@fata.unam.mx (R.Q.-T.); aragon@fata.unam.mx (J.L.A.); oca@fata.unam.mx (M.A.O.M.); 2 Department of Cellular and Molecular Neurobiology, Instituto de Neurobiología, Universidad Nacional Autónoma de México, Boulevard Juriquilla #3001, Juriquilla, Querétaro 76230, Mexico; fvazquez@comunidad.unam.mx; 3 Instituto de Física, Universidad Nacional Autónoma de México, Ciudad de México 04510, Mexico; cloro@fisica.unam.mx; 4 Cátedras CONACyT, Centro de Física Aplicada y Tecnología Avanzada, Universidad Nacional Autónoma de México, Juriquilla, Querétaro 76230, Mexico

**Keywords:** microfabrication, polymeric microchip, laser ablation, PDMS devices, low-cost fabrication tool

## Abstract

In this article, we show an alternative low-cost fabrication method to obtain poly(dimethyl siloxane) (PDMS) microfluidic devices. The proposed method allows the inscription of micron resolution channels on polystyrene (PS) surfaces, used as a mold for the wanted microchip’s production, by applying a high absorption coating film on the PS surface to ablate it with a focused low-power visible laser. The method allows for obtaining micro-resolution channels at powers between 2 and 10 mW and can realize any two-dimensional polymeric devices. The effect of the main processing parameters on the channel’s geometry is presented.

## 1. Introduction

Micromachining techniques to create polymeric microfluidic or microchip devices have received significant attention in recent years [1]. With the appropriate design, these controlled microenvironments are an essential tool for biological studies [2], including drug discovery [3], early diagnostic testing [4,5], cell differentiation [6], and others [7]. Two-dimensional polymers’ patterning enables the implementation of controlled planar microenvironments and for the obtention of non-flat surfaces [8] for cross-disciplinary research at the intersection of photonics, chemical, physics, nanotechnology, nonlinear fluid mechanics, and environmental science. Cross-disciplinary research has also benefited from polymeric microchip devices, which constitute tools for antiviral research and technical elements for treatment developments [9]. Requirements for microfluidic devices are well-established: scalability [10], reproducibility [11], and user-friendly characteristics to quickly move from lab-based prototyping to commercial manufacturing [12] are commonly specified. Typical microchips production may entail, for example, photolithography [13], microcontact printing [14,15,16], replica molding [17], or, as we show here, laser ablation. 

Because of the high patterning resolution, simple processing, and low-cost fabrication method, laser ablation has become an important tool to obtain polymeric microfluidic devices, highlighting poly(dimethyl siloxane) (PDMS) for studies in biological systems [18,19,20,21]. Frequently, a microfluidic system consists of a polymer-covered transparent glass plate in which a well-defined micro-channels and cavities pattern is engraved to provide an appropriate mechanical and chemical environment for a system of interest [22]. The single microchannel, through which fluids can flow, is the fundamental structure of these devices [23]. To shape such microstructures, pulsed or continuous laser ablation, usually assisted by a computer design software, can be used to direct-write a specified pattern. Material removal by laser ablation can be fitted or adjusted by the properties of the ablated material, by modifying the absorption characteristics of the material surface or by the laser properties [24]. The acceptable materials damage threshold for the laser ablation system determines the limits to energy exposure and the depth of focus on which the micromachining can work properly. Since the continuous laser can produce extensive material damage at the usual combination of material absorption and laser power, light pulses with widths in the nanosecond to femtosecond scale have been successfully used to better control the flow of energy to the ablated materials. Recently, a successful demonstration of laser micromachining of biodegradable materials, poly(glycerol sebacate) (PGS), poly(1,3-diamino-2-hydroxypropane-co-polyol sebacate) (APS), and poly(dimethyl-siloxane) (PDMS), with four nanoseconds pulse width and an ultraviolet (UV) wavelength laser of 193 nm, showed the widths of linear channels ablated on such PGS, APS, and PDMS using 10 μm beam size as 8, 11 and 12 μm, respectively [25]. The CO_2_-laser system provides a cost alternative to the UV-laser system and has demonstrated enormous potential for producing polymer microchips-based polyethylene terephthalate (PET), Poly(methyl methacrylate) (PMMA), polycarbonate (PC), polystyrene (PS), and enormous potential for producing PMMA microfluidic structures with laser beam power between 4.5 and 65 Watts, obtaining typical channel width between 60 and 300 μm [26,27,28,29]. However, long-wavelength lasers require expensive broadband infrared (IR) optics, a large size facility, and safety issues arise when no visible light is used because no warning due to the blinking reflex of the eye is possible [30]. Laser ablation with visible wavelength lasers represents a balanced option to reduce system cost, reduce risk to the users, and offer good resolution printing characteristics [31,32].

**Figure 1:**
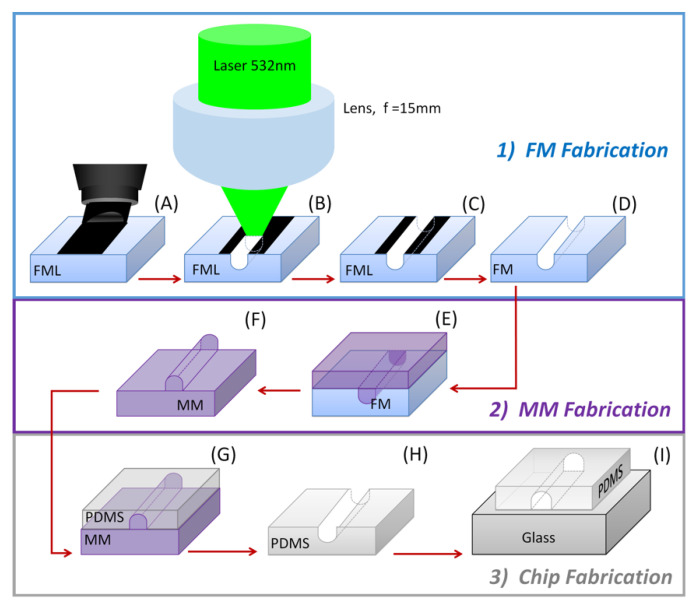
Fabrication method to obtain poly(dimethyl siloxane) (PDMS) devices with three main fabrication steps, (**1**) female mold (FM) fabrication, (**2**) male mold (MM) obtaining, and (**3**) chip assembling. (**A**) Layer film made on a face of polystyrene (PS) spectrophotometer cuvette by black sharpie marker (FML), (**B**) laser writing of the microchip features into the FML, (**C** and **D**) FM obtained by cleaning the ablated cuvette surface and cutting the imprinted side, (**E** and **F**) resin layer casting on the FM and peeled off to obtain a MM, (**G** and **H**) PDMS were cast on the MM and peeled off from it to get a polymeric chip layer, and (**I**) the final microchip is obtained by assembling the PDMS onto a glass slide.

In this article, we show an alternative strategy to obtain planar PDMS microfluidic devices [8] using a continuous low-power visible source for laser ablation microfabrication. This approach circumvents the use of expensive and complicated UV or IR lasers. The proposed laser ablation method is a low-cost, simple procedure to obtain PDMS microfluidic devices. It consists of three main steps: (1) female mold (FM) fabrication, (2) male mold (MM) obtaining from FM, and (3) chip fabrication by PDMS cast on the MM, peeling from it, and final bonding onto a glass slide. In our case, green laser ablation is used onto a side of polystyrene (PS) cuvette for conventional spectroscopic use, with a female mold layer (FML) as the FM, we used an epoxy resin as a MM to replicate PDMS devices, and PDMS cast on such MM, which is peeled off to finally bond it onto a glass slide, as shown in Figure 1. This reachable system is a simple optics and low-cost tool based on accessible materials and methods. On the one hand, the microfabrication presented might be useful to study methods needed for prototyping microchannel devices using other polymers. Still, PDMS has maintained its popularity due to gas permeability for cell culturing, high-fidelity reproduction, and optical transparency, e.g., rapid fabrication of microfluidic PDMS devices from reusable PDMS molds have also been used [29]. The visible ablation process on PS presented here offers several advantages. It produces micron channel resolution with a very low-intensity power of continuous laser centered at 532 nm visible wavelength. These microfluidic devices obtained with the proposed method allow the production of high-resolution, precisely aligned, and homogeneous channels. Besides that, the method can be easily adapted for the microfabrication of two-dimensional features on a polymeric material, see, e.g., [25].

## 2. Materials and Methods 

Following the schematics in Figure 1, the micromachining of a microfluidic device begins with the fabrication of a predesigned female mold onto a side of a previously washed polystyrene cuvette for conventional spectroscopic use. The selected face of the cuvette is hand-drawn overcoated using a permanent marking pen [33] with a rapid marker masking technique [34] to increase its surface optical absorption, as suggested in a laser ablation procedure [35]. In our case, a thin layer film of permanent ink marker (Sharpie Magnum black permanent ink marker) was used to hand-draw a thin layer film of ink onto one side of a polystyrene sample to reduce the damage threshold. The ink of these permanent markers contains resin, a carrier solvent, and a pigment to promote adhesion [36], and can be easily removed with a proper solvent [37]. The so-prepared cuvette is mounted on a computer-controlled three linear axis motorized stage (Thorlabs MTS50) that displaces it according to the programmed set of instructions to imprint the microchip features on the cuvette surface in a highly reproducible way. 

**Figure 2:**
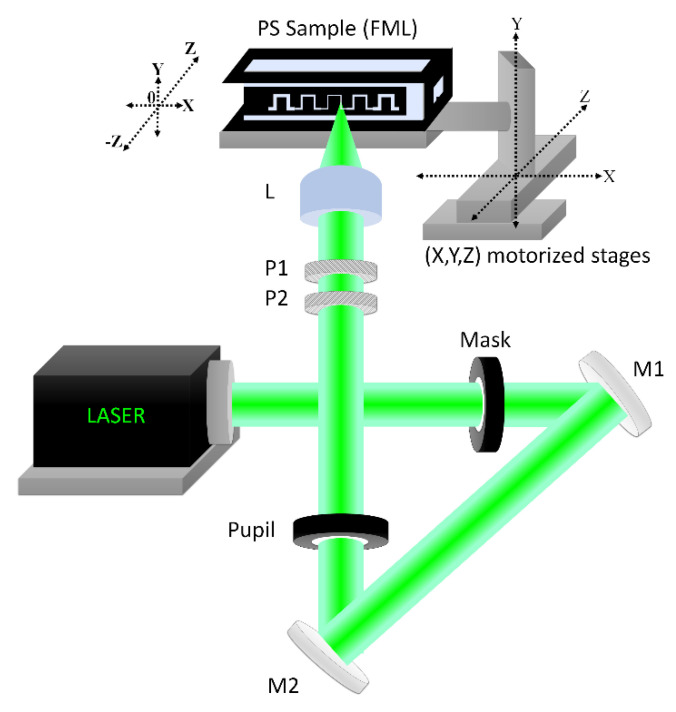
The optical system imprints the microchip features on a surface of a polystyrene cuvette. A green laser beam with an initial profile is rectified by a circular Mask. Two mirrors (**M1**, **M2**) redirect the beam toward the FML supporting stage. A circular pinhole filters the central part of the beam from their diffracted components as a Pupil. Two linear polarizers (**P1**, **P2**) set a convenient beam polarization. A plano-convex lens (**L**) focuses the beam at the surface of the FML. Three linear motorized stages (X, Y, Z, Thorlabs MTS50) are used.

The ablating system, as seen in the schematics of Figure 2, consists of a continuous frequency-doubled Nd:YAG laser, pumped by a solid-state diode to give a wavelength centered at 532 nm. The optical system brings such 532 nm light onto the FML. A circular Gaussian beam profile is given to the laser beam by passing it through a pinhole-type circular mask. Light diffraction in the far-field passing this mask is observed as the Airy’s rings associated with this circular aperture. The laser profile might be changed to another type as desired for other applications by simply changing the mask shape [38]. After passing the mask, the beam is deviated by two flat mirrors (M1, M2), used to reduce the overall setup dimensions, redirecting it toward the support cuvette. Before allowing beam incidence on the ablating surface, it is passed through a second pinhole, used as a pupil to remove its second and higher diffraction orders, and then through a set of two perpendicularly oriented linear polarizers (P1, P2), which control the polarization and tune the average beam power on the ablated surface. By following Malus’s law, we control the maximum intensity of such transmitted linear polarized light. Finally, the beam passes through a 15 mm focal length of an uncoated plano-convex lens that focuses it on the surface to be ablated in such a way that the maximum intensity sharp circular image lays over the central part of the Z displacement of the ablating surface. The light intensity passing to the lens is diffraction-limited, and this optical setting largely reduces the noise in the beam intensity distribution, while it maximizes the ablating beam power. The overall numerical aperture of the system is basically determined by the coupling between the pinhole pupil and the focusing lens.

**Figure 3:**
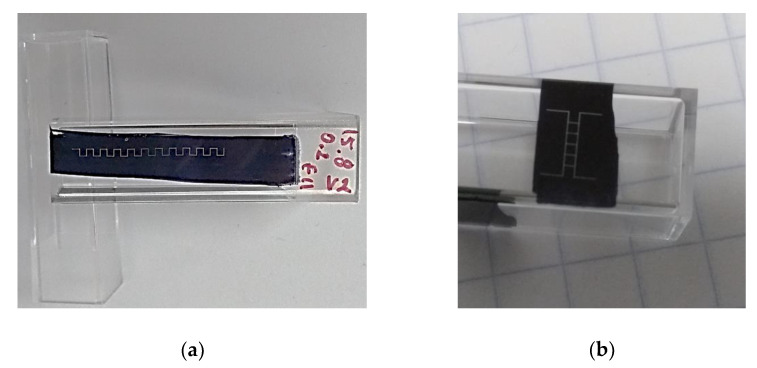
Micromachining by green laser ablation method. Two examples of FMLs, (**a**) and (**b**), obtained onto one side of the polystyrene spectrophotometer cuvette. (**a**) FML sample with different focal planes and (**b**) focused on a plane coincident with the ablated surface (Z = 0) (see Supplementary Information, Top view of sample and PDMS microchip Model 1).

Once the cuvette is fixed on the stage and the optical system is properly aligned onto its selected surface to ablate, laser beam exposure is allowed. Figure 3 shows two examples of an ablated cuvette surface, from which the FML is then obtained by cutting the inscribed plate from the whole cuvette. Different designs of models can be prepared in a similar way, just limited by the area of the layer film of permanent ink marker (see Supplementary Information, PDMS Microchannels Fabrication examples: PDMS microchip Model 1 and Microchannels Model 2). The obtained FML is then washed by cleaning the black marked surface with a solvent for 15 min in an ultrasonic isopropanol bath three times, rinsed in distilled water, and dried. Inlet and outlet ports can be drilled on marks made for that purpose during the FM production process. Most of the samples have suitable quality sidewalls, but sometimes there is static adhered debris caused by drilling such ports. In such cases, to further improve its quality and rescue the sample, the obtained FM can be exposed to alternate one-minute air and one-minute vapors of acetone five times, followed by isopropanol washing to remove such adhered debris. In that case, it is important to consider that the acetone vapor also affects the dimension of the FM mold while removing the debris, and one extra analysis should be considered. We recommend drilling the inlet and outlet ports before ablating the FML. Once obtained, the FM is put in a clean wide-open container to make the MM by pouring a commercial epoxy resin (Barniz Policromo^®^, two-component epoxy system A:B) onto it, placing it in a desiccator with a partial vacuum for 20 min to eliminate dissolved bubbles, and to a heated plate at 35 °C for 10 min to complete the resin setting. Once cooled down, the fixed resin was gently peeled off from the FM to obtain the MM, which is then used as a master mold to produce several PDMS films. The procedure to obtain the PDMS films, to finally make the wanted microchips, is similar to the one used to obtain the MM. The master MM is put in a wide-open container onto which a two-component resin (SYLGARD 184^®^) with a 10:1 mix ratio is poured. The container is then placed in a desiccator with a partial vacuum for 40 min and later transferred to a heated plate at 35 °C for 10 min. Once cooled, the PDMS film is peeled off from the master MM and bonded to a glass slide previously cleaned by washing it in an ultrasonic isopropanol bath for 15 min, followed by 15 min rinse in methanol, washed with milli-Q water, and dried in clean airflow (see Supplementary Information, PDMS Microchannels Fabrication examples: PDMS microchip Model 1 and Microchannels Model 2). 

## 3. Results

As mentioned in the last section, the two linear polarizers in the system allow good control of the ablating beam power. Even if P1 and P2 are removed, ablation is possible; however, a lower sidewall quality of the channels is commonly observed in this case. We noticed that with simple exposure to light illumination, photoablation is produced on the FML with a relatively low average power. Defects are also common when non-uniform blackening of such FML is achieved. Beam powers above 10 mW also result in rough channel sidewalls and potential material burning.

**Figure 4:**
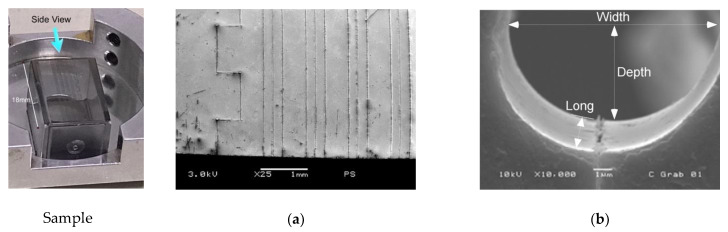
FM sample is obtained onto one side of the polystyrene spectrophotometer cuvette of 10 × 18 mm^2^, using a speed of 0.02 mm s^−1^, and average power of 9.3 ± 0.1 mW, focused on a plane coincident with the ablated surface (Z = 0). The (**a**) shows a scanning electron microscope image of a top view of a polystyrene (PS) cuvette for conventional spectroscopic use. (**b**) Scanning cross-section image of an 18 mm long microchannel.

A scanning electron microscope image of an 18 mm long, 12 μm width, and 6 μm depth microchannel, run at a stage speed of 0.02 mm s^−1^ and an ablating power of 9.3 ± 0.1 mW is shown in Figure 4b (see Supplementary Information, Scanning Electron Microscopy Analysis: Top view of sample and Side view). Well-defined sidewalls and uniformity were observed. 

**Figure 5:**
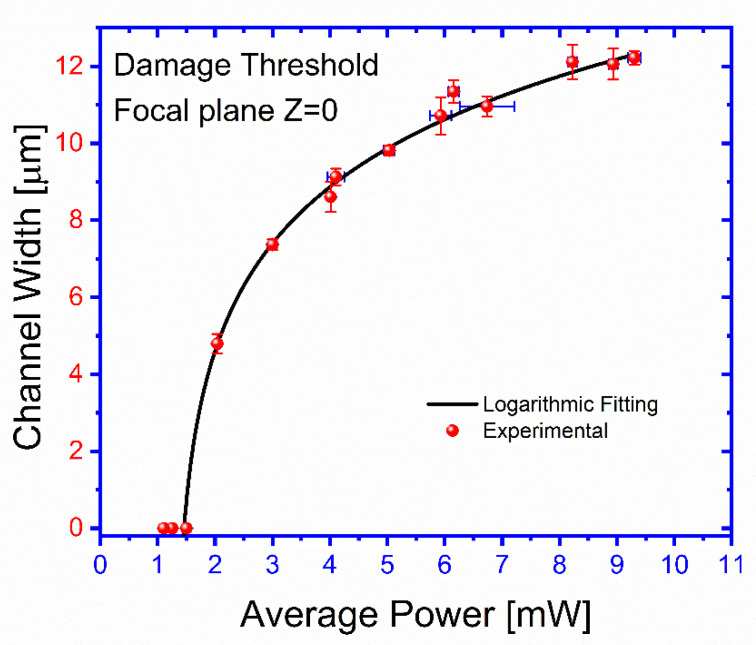
Laser micromachining channel width as a function of the beam average power for a beam focused on the ablated surface (Z = 0). The laser-induced damage threshold is about 2 mW. For a beam power smaller than this value, no ablation marking was observed.

Figure 5 shows the printed channel width as a function of the Gaussian beam average power for a beam focused on the ablated surface Z = 0. First, a low-power beam is directed to the surface of the selected face of the cuvette that is hand-drawn overcoated using a permanent marking pen with a rapid marker masking technique. The surface is exposed in different locations to this laser beam using a speed of 0.02 mm s^−1^, and average power of 9.3 ± 0.1 mW, focused on a plane coincident with the ablated surface. After exposure, the surface is exanimated by microscope 40× magnification for any visible ablated channel (see Supplementary Information, Laser micromachining channel width as a function of the beam average power). The channels that are ablated at a particular power are recorded, and the diameter of the channel is measured. Next, the power is increased, and the surface is exposed at different new locations. According to the test, a power threshold damage of about 2 mW is evidently observed, and a smooth increasing dependence of the channel width with the beam power is also seen. 

**Figure 6:**
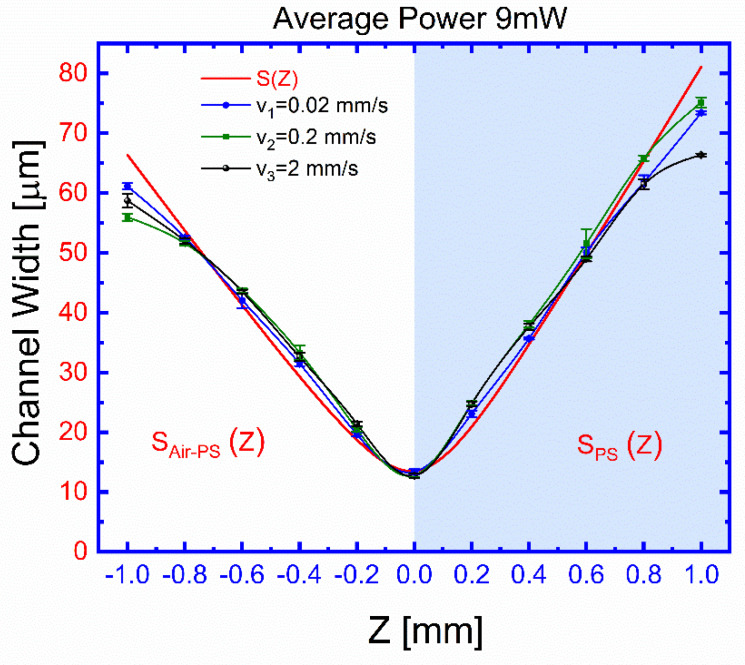
Channel width (S) as a function of the focal plane distance to the ablated surface (Z) for different transversal (X or Y) speed displacements: $v_{1}=\;$0.02 mm s^−1^ (blue circles),$\;v_{2}=\;$0.2 mm s^−1^ (green circles), and $v_{3}=\;$2 mm s^−1^ (black circles). *Z* > 0 for the beam focused within the ablated material, and *Z* < 0 for the beam focused before the FML surface. The Gaussian fitted beam diameter as a function of Z in Equation (1), in red, correlates well with the observed channel width for the explored displacement velocities for |Z|<1 mm.

A more pronounced dependence of the channel width on the distance Z between the ablated surface and the focusing plane of the beam, for different transversal displacement (X or Y) speeds, is shown in Figure 6, which also shows the dependence with Z of the beam diameter at the ablated surface. Data for Figure 6 were taken from different planes, orientations, and sample surfaces at an average beam power of 9 mW, and the three experimental curves are taken at different speeds of different samples. The velocities of the translation stage are: 0.02 (blue circles), 0.2 (green circles), and 2 mm s^−1^ (black circles). For each curve as a function of distance Z, the surface is exposed in different locations to this laser beam using the selected velocity of the translation stage, and the average power of 9 mW is fixed. After the exposure for different transversal displacement (X or Y), the distance Z is increased or decreased, and the surface is exposed at different new locations. After exposures, the surface is examined by microscope 40× magnification for any change in the channel width (see Supplementary Information, Channel Width S and Laser micromachining channel depth). The channel width that is ablated at a particular Z and speed is recorded.

From Figure 6, our printed semi-circular channel’s width as a function of distance Z between the ablated surface and the focusing plane is a cross-section estimated over the beam’s full width at half its maximum intensity (FWHM) used by the visible laser. The beam diameter S is estimated (Figure 6, red line) by fitting a Gaussian profile [39] to the beam near the focusing plane by the following expression:

(1) $$S\left(Z\right)=S_{0}\sqrt{1+{\left(\left(\frac{n_{eff}}{n_{0}}\right)\left(\frac{\lambda \;Z}{\pi \;S_{0}{}^{2}}\right)\right)}^{2}}\;,$$

where, $S_{0}$ is the beam diameter at the focal plane *Z* = 0, λ=532 nm is the beam wavelength, $n_{0}\;\approx \;1$ is the air index of refraction, and $n_{eff}$ is the effective index of refraction at the beam focusing position, which is taken as polystyrene index, $n_{PS}\;$= 1.5983 for the beam focused within the ablated material (*Z* > 0, condition $S_{PS}\left(Z\right)$), and fitted as an average $n_{Air-PS\;}$= 1.2991 for a broad interface between air and polystyrene (*Z* < 0, condition Air–PS, $S_{Air-PS}\left(Z\right)$).

**Figure 7:**
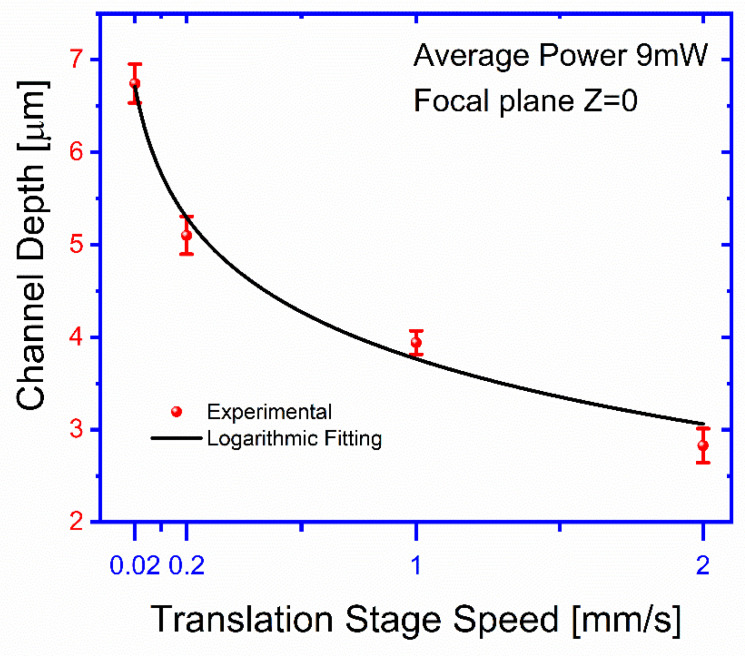
Channel depth as a function of different translation stage speed. The speed of the translation stages changes the depth of the channels’ range, from 2.8 ± 0.18 to 6.7 ± 0.2 μm, and the logarithmic approximation using Equation (2) (black line).

The channel depth, d, as a function of the stage translation speed, v, was also obtained for an ablated beam power of 9 mW at the focal plane. For each curve, as a function of speed, v, the surface is exposed in different locations to this laser beam using the selected velocity of the translation stage, and the average power of 9 mW is fixed. After the exposure for different transversal displacement (X or Y), the speed, v, is increased, and the surface is exposed at different new locations. After exposures, the surface is examined by microscope 40× magnification, and optical depth measurements are taken by focusing the upper and lower surfaces of the channel and reading off the travel of the tube of the microscope by means of the calibration of the fine adjustment knob. The results presented in Figure 7 show a change in the depth of the channels from 2.8 ± 0.18 to 6.7 ± 0.2 μm for a change of 2 orders of magnitude in the translation speed. The obtained results can be approximately fitted by the following relationship:

(2) d(v)=A−B∗ln(v+C),

where A=3.81±0.24, B=1.04±0.4, and C=0.04±0.08. 

**Figure 8:**
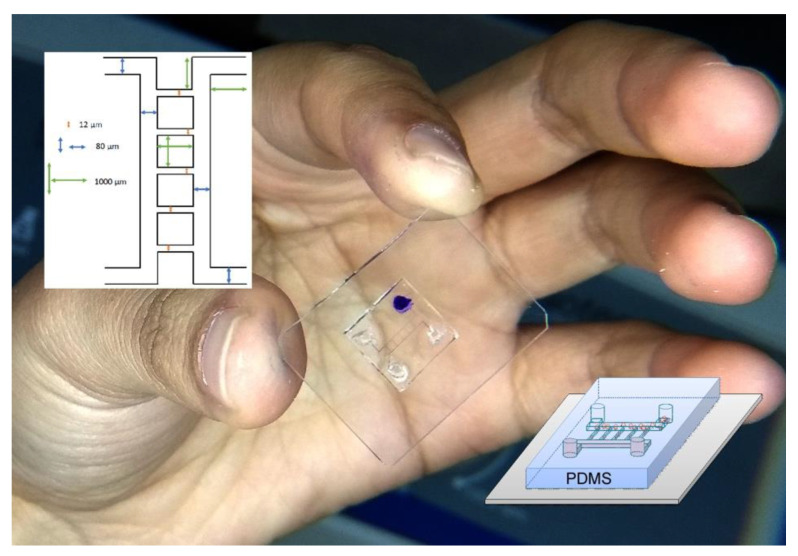
PDMS microchip device. The microchip consists of a poly(dimethyl siloxane) (PDMS) microfluidic device on the top of a glass substrate. This structure supports an alternative low-cost fabrication method with which a low-power visible ablation is possible. The insets show a schematic view of our microchip.

One result of our alternative low-cost fabrication method to obtain our final poly(dimethyl siloxane) (PDMS) microfluidic device is shown in Figure 8. A solvent with crystal violet dye was prepared to observe the experimental channels and the insets show the schematic view of the microchip (see Supplementary Information, PDMS Microchannels Fabrication examples: PDMS microchip Model 1 and Microchannels Model 2).

## 4. Discussion

The main observed result concerns the feasibility to obtain width and depth controlled microchannels by an ablation process with a very low-power visible laser. Micron resolution and good surface quality can be obtained for laser powers in the range from 2 to 10 mW for a 532 nm wavelength laser. Such feasibility is highly promoted by the use of a blank ink as an absorptive coating on the FML, which also has an important role in the quality of the ablated surfaces. 

The results shown in Figure 5 and Figure 7 show the effects of the processing parameters on the geometrical characteristics of the ablated channels. Beam focusing is the most influencing factor for the channel widths when a micron resolution is desired. This might be understood in terms of the relatively low power threshold required to produce damage for the considered FM material, which under a wider unfocused beam can ablate a larger area. A rough estimate from the results in Figure 6 suggests that control channel widths within a micron precision, the relative displacement of the focal plane, and the ablated surface, should be below 0.1 mm over the inscribed region. This estimation sets the importance and limits to the flatness of the FM surface and to its proper alignment on the supporting stage. A slight asymmetry, to be considered for good control of the process, is also observed for the channel width dependence on Z. This can be explained by the change of the beam geometry as we move the focusing plane from the outside of the ablating surface to its interior. On the other hand, practical independence of channel depth, which changes by a factor of 2.3 for two orders of magnitude change of the displacement velocity, seems to indicate that once a superficial ablation has been attained, the effectiveness to digging increase is attenuated. The obtained results suggest that, even though the deepening process is larger for smaller translation velocities, it seems to not be as efficient as the widening one. This may indicate that on losing the blackening film on the FML surface, the ablating process becomes highly reduced.

Fabrication of microchannel via laser ablation is created with a simple lithography tool to direct writing on the FML surface. We used two spatial filters with pinholes as a circular mask and a pupil to improve the laser quality of a Gaussian beam and two linear polarizers to easily control the average power. Two metallic mirrors before a lens have been used to obtain a compact micromachining tool with the help of three automatic translation stages. A thin-layer film of permanent ink marker was deposited onto one side of a PS cuvette with a rapid marker masking technique. It is important to note that a defect-free line of permanent ink marker is convenient for laser ablation. Defects are present typically at the edge of the film when the line is produced by being hand-drawn directly onto the PS surface [37]. The uniformity of this ink film is a determining factor to produce defect-free channels.

We study the relative lowest light dosage and the changes through the focal plane to find the nominal best laser ablation condition. A basic correlation between the channel width and the beam diameter, which might be expected from the fact that such diameter encloses 86% of the beam power, is clearly observed. A slight asymmetry, to be considered for the good control of the ablation process, is observed for the channel width dependence on Z due to the beam geometry change as we move the focusing plane from the outside of the ablating surface to its interior. 

The laser-induced damage threshold is more than 2 ± 0.04 mW, and below this level of average power, the FML is not ablated. As shown in Figure 5, the channel limits obtained at the focal plane were within an exposure intensity dose range 2 ± 0.04 to 9.3 ± 0.1 mW, corresponding from 4.7 ± 0.24 to 12 ± 0.17 μm. When the average power is above 10 mW, it results in less smooth channel sidewalls and burns the FLM. Also, we found that it can stay within such exposure dose range, but it can be ablated if it remains within a particular focus range to print channels. As shown in Figure 6, the channel width achieves its best focus at such a focal plane Z = 0. In either ±Z-direction, both curves show that the channel gradually grows out of focus, similar to the calculated channel widths $S_{PS}\left(Z\right)$ and $S_{Air-PS}\left(Z\right)$. The area of the beam is, therefore, directly proportional to the size of the channel width. An experimental channel width was obtained using 9.3 ± 0.1 mW of average power and by changing the distance at the focal plane range from 12 ± 0.17 to 75 ± 0.85 μm. Figure 7 shows the channel depth as a function of different translation stage speed. The depth of microchannels is controlled using the speeds of the translation stages. In this case, we observe that the stage’s speed changes the depth of the channel range from 2.8 ± 0.18 to 6.7 ± 0.2 μm. The comparison between the results shown in Figure 6 and Figure 7 suggests that even the deepening process is larger for smaller translation velocities, it seems not as efficient as the widening one, which might suggest that once surface blackening is lost during a first ablation stage, further material removal becomes ineffective. Therefore, the precise control of the geometry is obtained by adjusting the laser beam’s speed and average power.

Our fabrication method based on green laser ablation to obtain microchannels is demonstrated, and regardless of the low cost, our tool has considerable implications. First, a thin-layer film of ink markers deposited onto one side of a polystyrene substrate will enhance visible laser ablation, so a user-friendly low-intensity power of visible light for microchip fabrication is possible; likewise, our tool also matches the dependence of the channel width (S) and the precise control of the geometry is obtained by adjusting the speed of the translation stage and the average power of the laser beam [40]. When a larger channel width is needed, the laser machining process is performed using a focused and unfocused laser beam [41] governed by Equation (1). Second, our method is a simple tool consisting of three main fabrication steps, female mold (FM), male mold (MM) used to replicate PDMS devices, and chip fabrication [29], but neither CO_2_ nor pulsed laser is needed. Compared with other infrared laser ablation methods with more expensive and complex laser systems, the resolution obtained is improved [8]. 

## 5. Conclusions

In conclusion, our ablation system using a visible wavelength laser represents a balanced option to reduce system cost, reduce risk to the user, and offer good resolution printing characteristics. The obtained results demonstrated that a low-power visible ablation is possible to obtain high-resolution microchannels in polymeric materials by enhancing the optical absorption of the ablating surface with a blackening simple commercial ink. The presented system is a simple optics and low-cost tool based on the use of simple materials and methods, in which simple controlled parameters like the beam power and the displacement velocity of the ablated surface determine the quality and geometrical characteristics of the obtained microchips. The visible ablation process presented here on a PS surface has shown the feasibility of producing microchannel resolution by using a very low-power continuous laser in the interval from 2 to 10 mW, centered at a green 532 nm wavelength. Such a process is a cost-effective and alternative strategy to fabricate PDMS devices. Fundamental cross-disciplinary research has demonstrated the importance of polymeric microchip devices, providing tools and technologies for antiviral research and treatment development [9]. The choice of the green ablation micromachining technique to create a polymeric microchip bonded on a glass slide shows that the method is not limited to biologically relevant samples and may be implemented on the ongoing SARS-CoV-2 pandemic. Therefore, possible application in two-dimensional patterning enables the implementation of controlled planar PDMS microenvironments and also possible applications on non-flat surfaces for cross-disciplinary research at the intersection of photonics, chemical, physics, nanotechnology, nonlinear fluid mechanics, and environmental science. The microfabrication presented here might be useful to study methods needed for prototyping microchannel devices with other polymers.

## Data Availability

Data is contained within the article or supplementary material, correspondence and requests for materials should be addressed to J.L.D.-J. (jldominguezju@conacyt.mx).

## Supplementary Materials

The following are available online at https://www.mdpi.com/2073-4360/13/4/607/s1. Scanning Electron Microscopy Analysis: Top view of sample, Side view. PDMS Microchannels Fabrication examples: PDMS microchip Model 1, Microchannels Model 2. Supplement Information of Figures: Laser micromachining channel width as a function of the beam average power, Channel Width S, Laser micromachining channel depth.
