# Supplementary material for: Microfabrication with Very Low-Average Power of Green Light to Produce PDMS Microchips
Source: Polymers (Basel). 2021 Feb 18;13(4):607. doi: 10.3390/polym13040607 (PMC7921959; doi:10.3390/polym13040607)
Supplement: Supplementary file 1 [file polymers-13-00607-s001.pdf]

SUPPLEMENTARY INFORMATION

1. Scanning Electron Microscopy Analysis 2

Top view of sample. 2

Side view 2

2. PDMS Microchannels Fabrication examples. 3

PDMS microchip Model 1 3

Microchannels Model 2 4

3. Supplement Information of Figures 5

Laser micromachining channel width as a function of the beam average power. 5

Channel Width S 9

Laser micromachining channel depth 15

1. Scanning Electron Microscopy Analysis.

Top view of sample.

Scanning electron microscope images of multiple channels obtained onto one side of 10mmx18mm polystyrene spectrometer cuvette, run at a stage speed of 0.02mms^{-1} and an ablating power of 9.3±01mW are shown in A) and different areas of the sample B), C), D), E) and F).

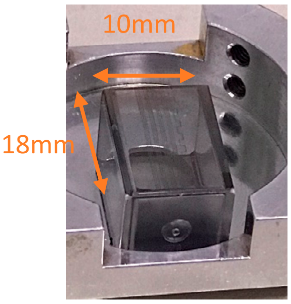

1.A)

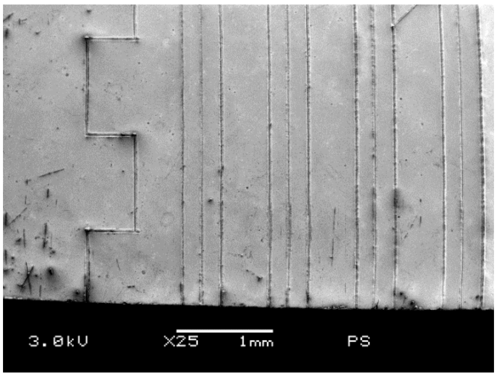

1.B)

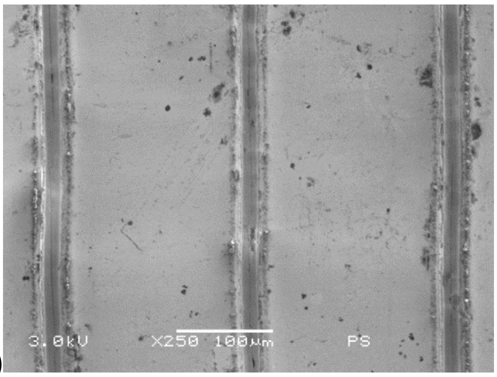

1.C)

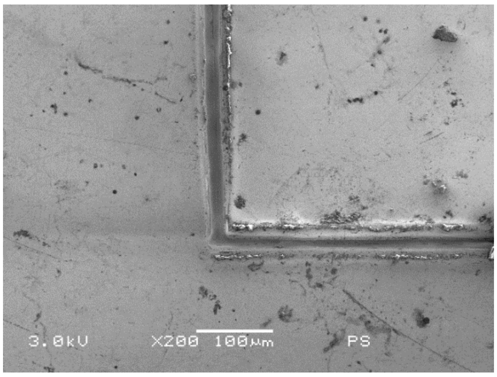

1.D)

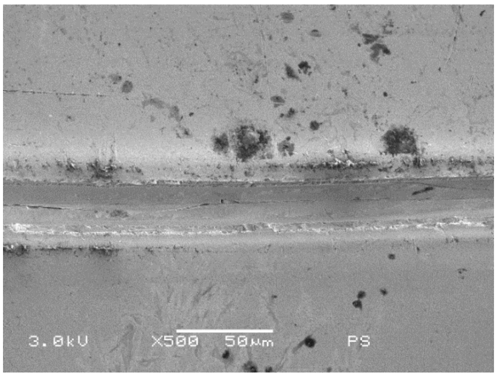

1.E)

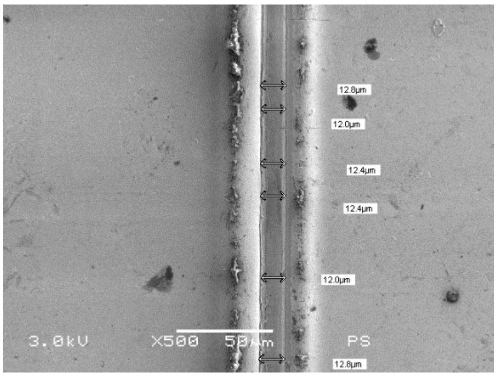

1.F)

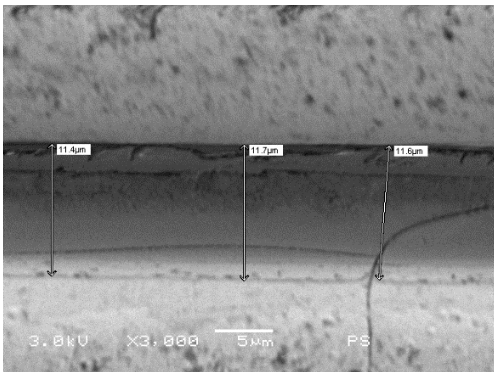

Side view

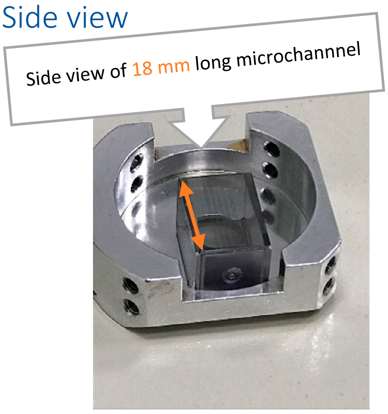

Scanning Electron Microscope cross-section image 1.1.G) of 18mm long microchannel on a FM focused on a plane coincidednt with the ablated Surface (Z=0).

1.1.G)

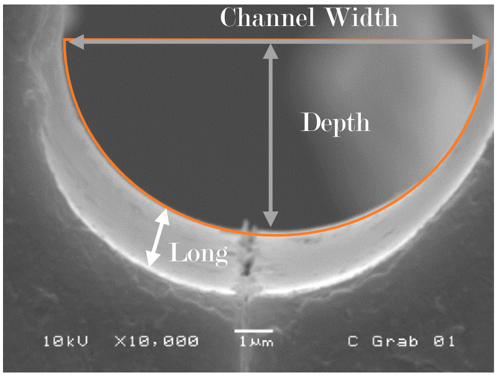

2. PDMS Microchannels Fabrication.

PDMS microchip Model 1

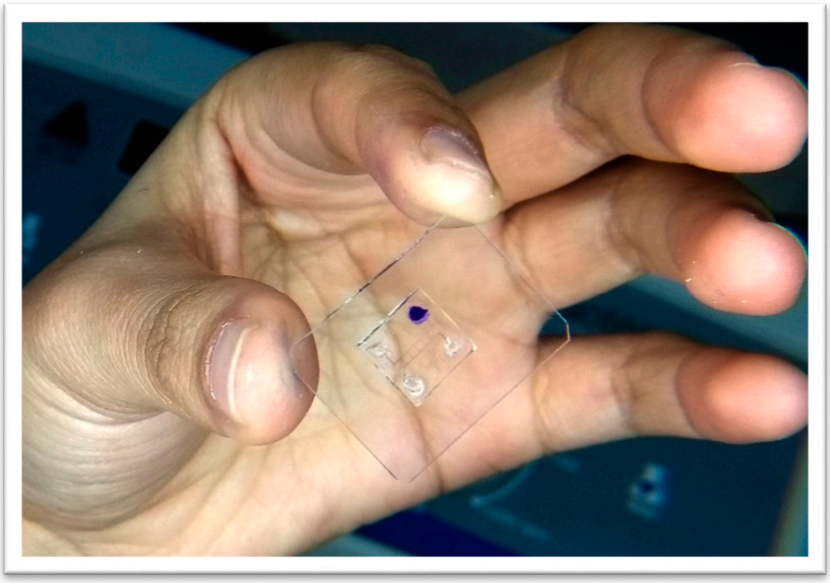

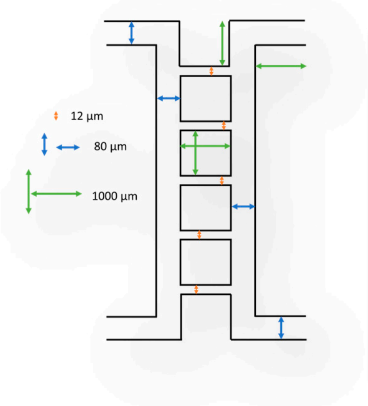

2.1.A) Diagram of model 1.

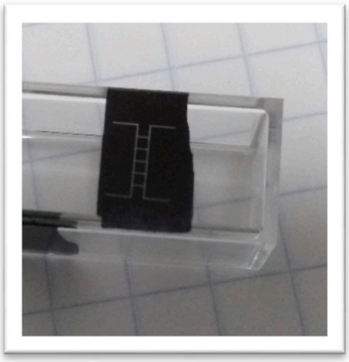

2.1.B) FML of Model 1

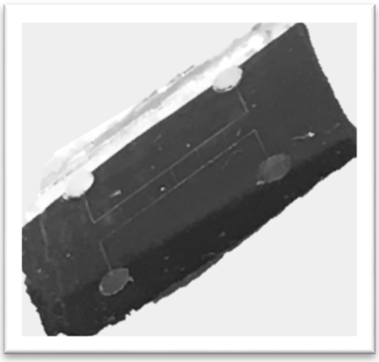

2.1.C) FML with ports drilled.

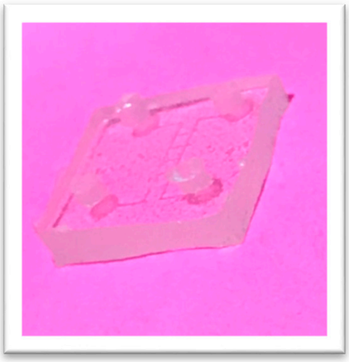

2.1.D) Male Mold of M1

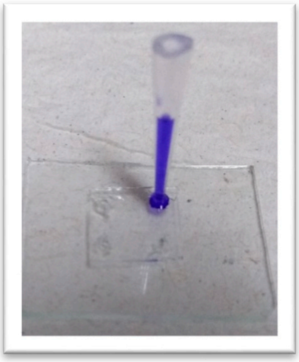

2.1.E) PDMS chip with fluid (crystal violet dye).

PDMS microchip Model 2

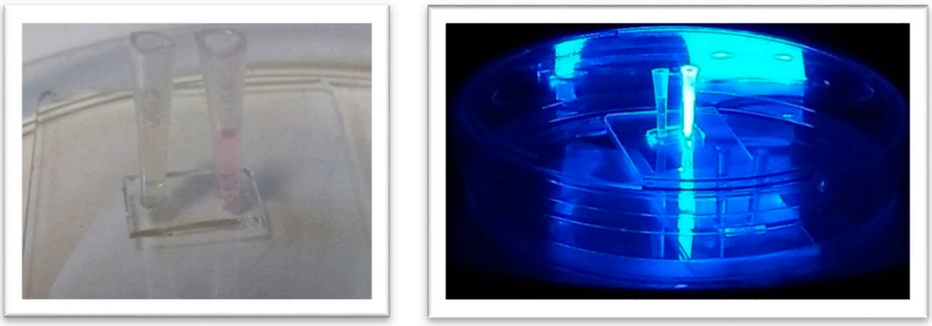

CellTracker experiment using fluorescent dye for monitoring cell movement or location.

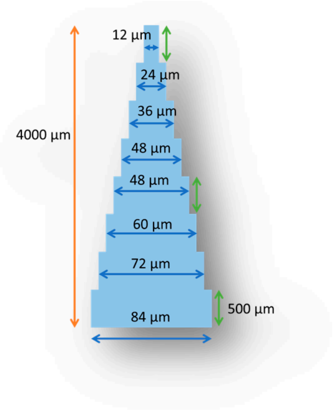

2.2.A) Diagram of model 2.

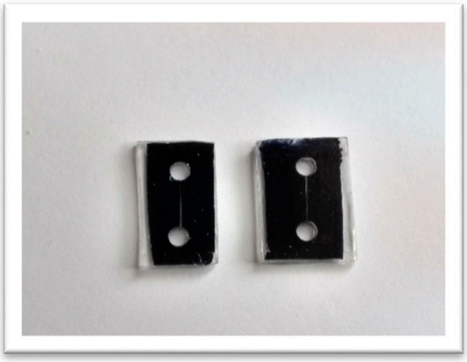

2.2.B) FML of model 2 with ports drilled.

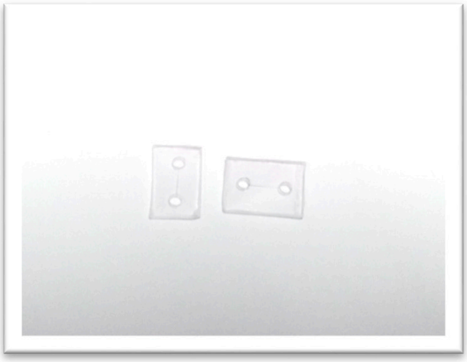

2.2.A) FM of model 2.

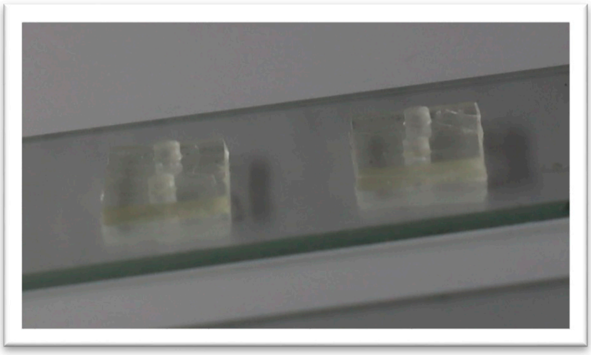

2.2.B) MM of model 2

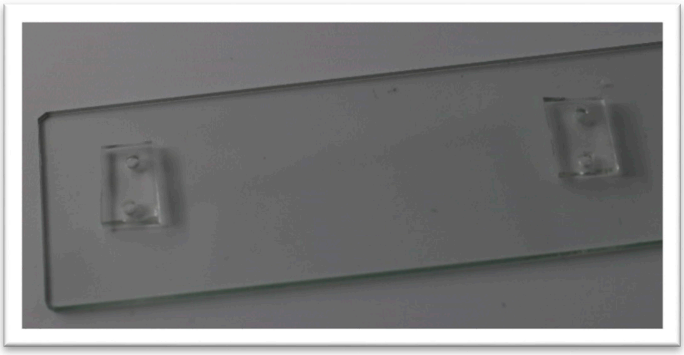

2.2.A) PDMS chips of model 2

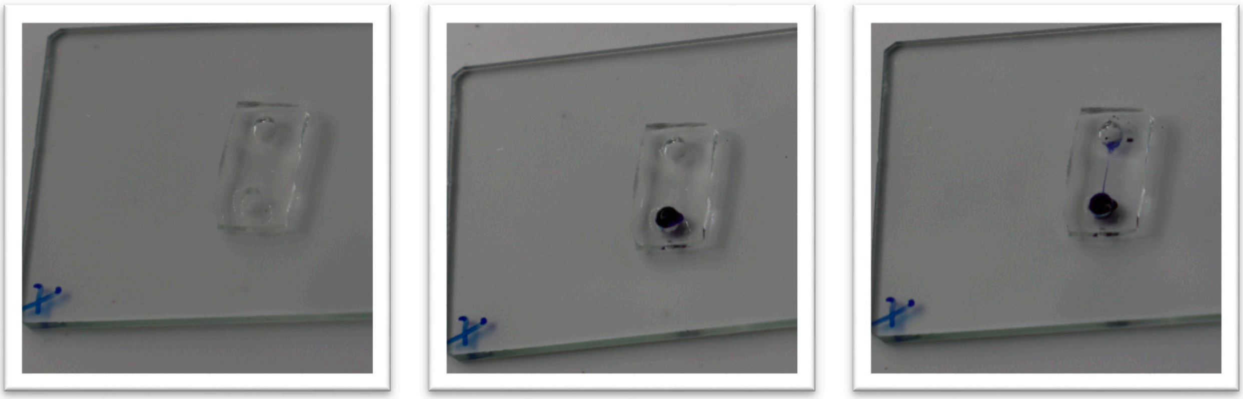

2.2.E) PDMS chip with fluid (crystal violet dye).

3. Supplement Information of Figures

Laser micromachining channel width as a function of the beam average power.

Channel width as a function of the beam average power at the focal plane distance to the ablated surface Z=0. The surface is observed by microscope 40x magnification and optical width measurements are taken for different values of beam power.

Table of channel width [row Beam Power: (column Measure 1, column Measure 2)].

Beam Power	Measure 1	Measure 2
1mW	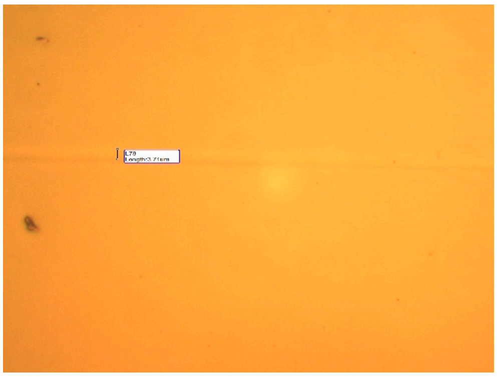	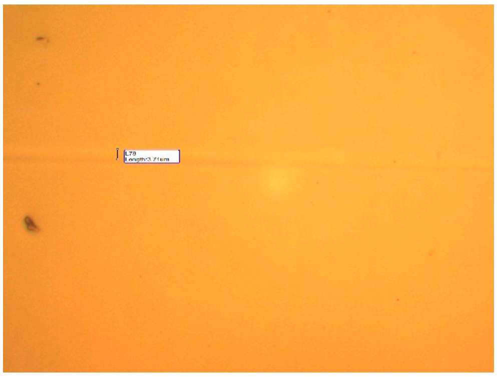
1.5mW	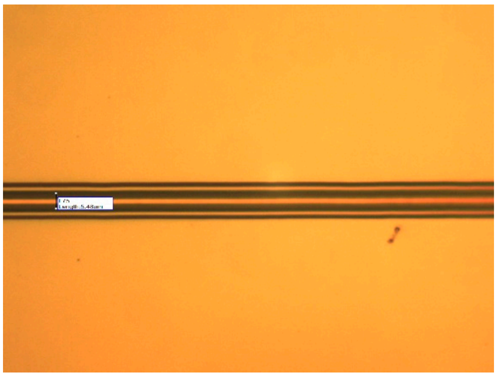	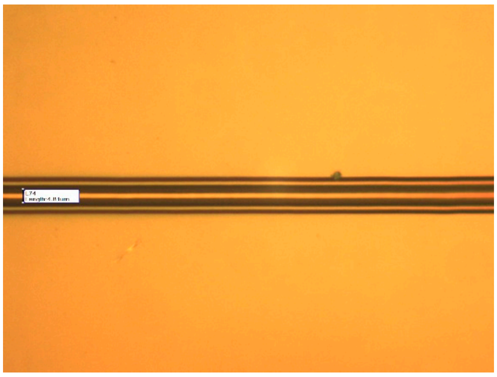
2mW	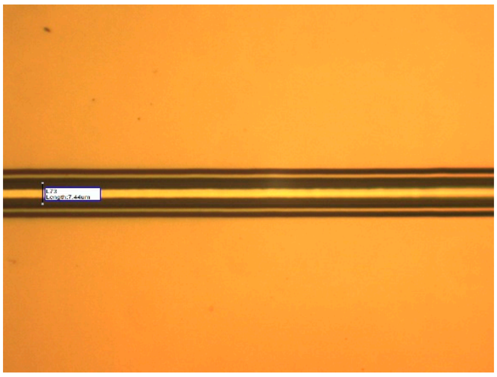	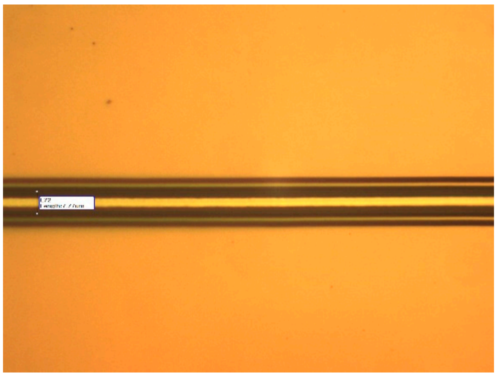

3mW

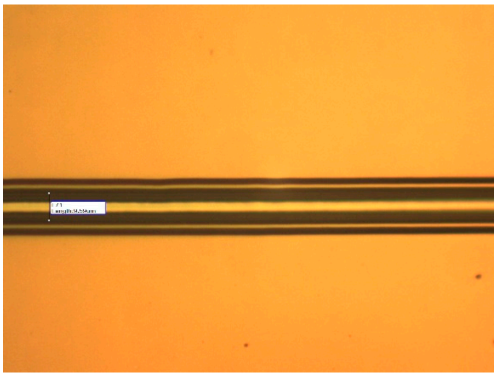

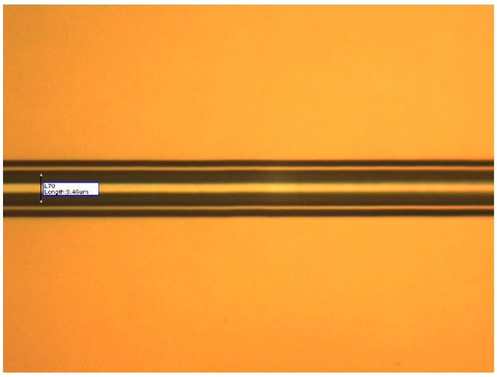

4mW

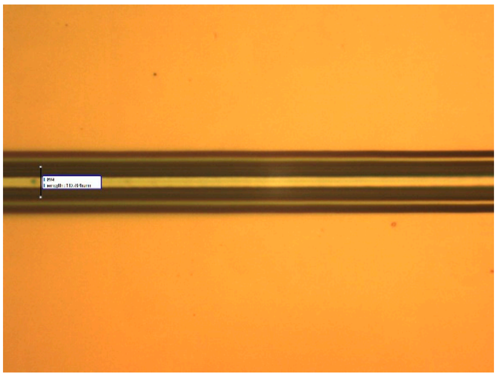

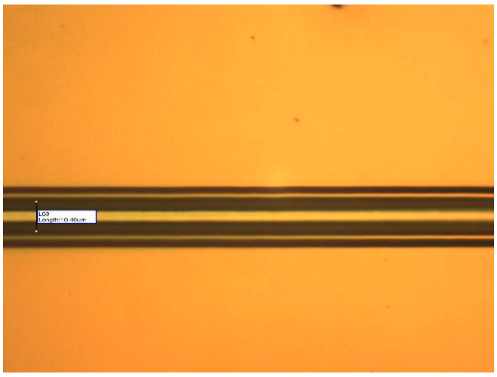

5mW

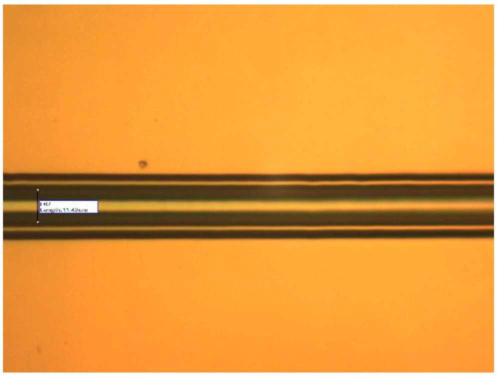

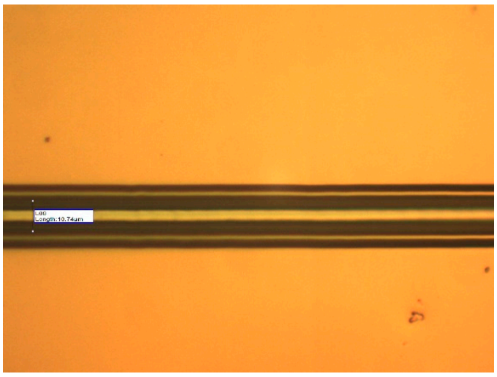

6mW

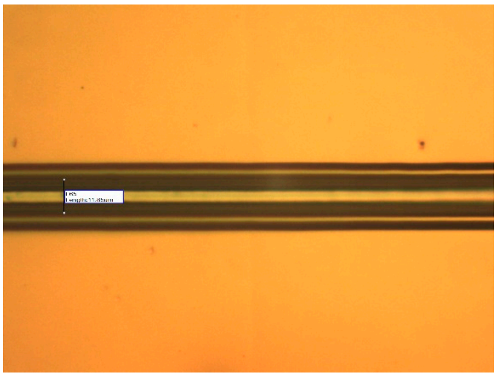

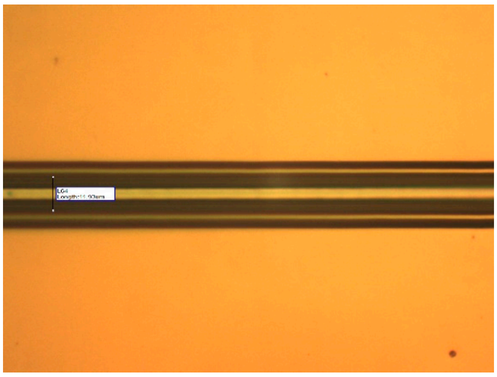

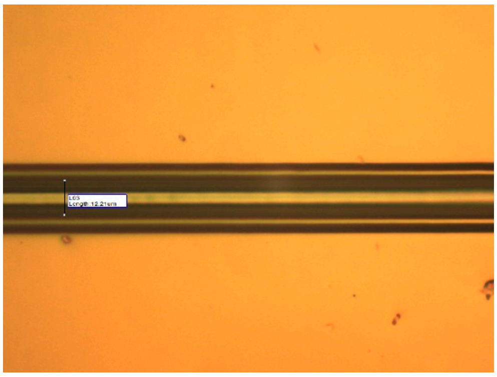
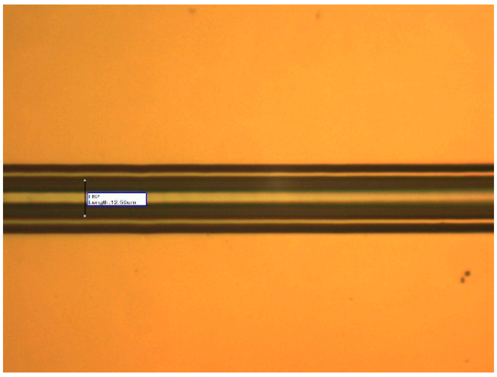

7mW

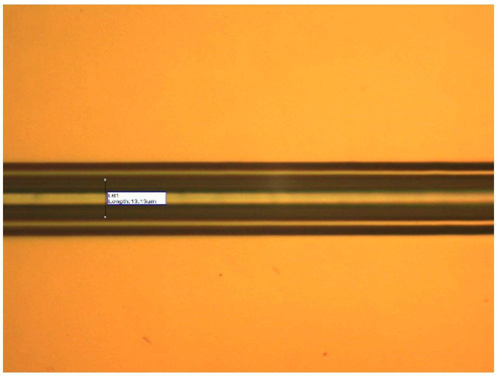
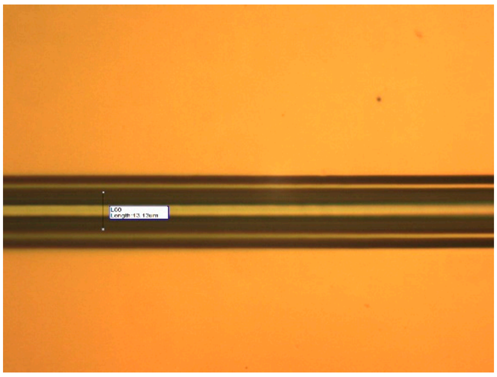

8mW

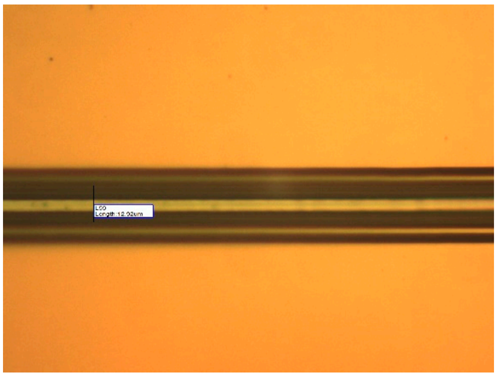
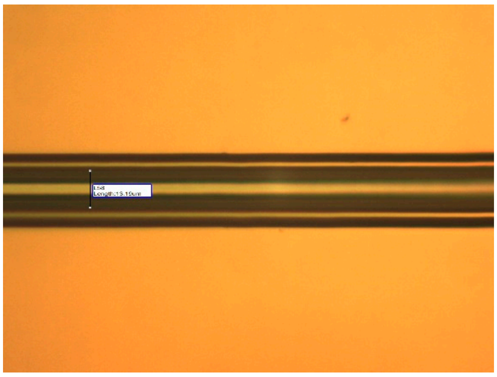

9mW

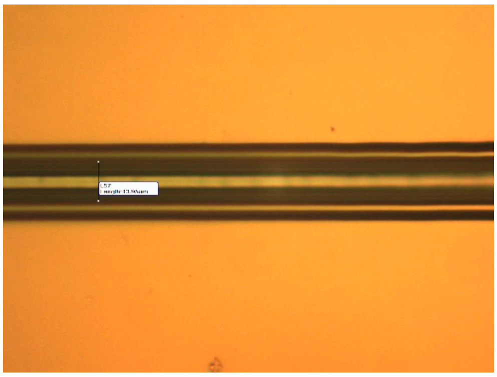
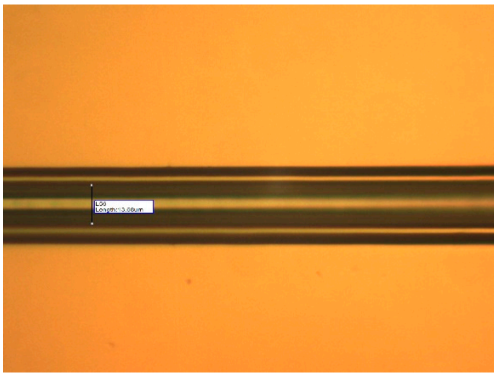

10mW

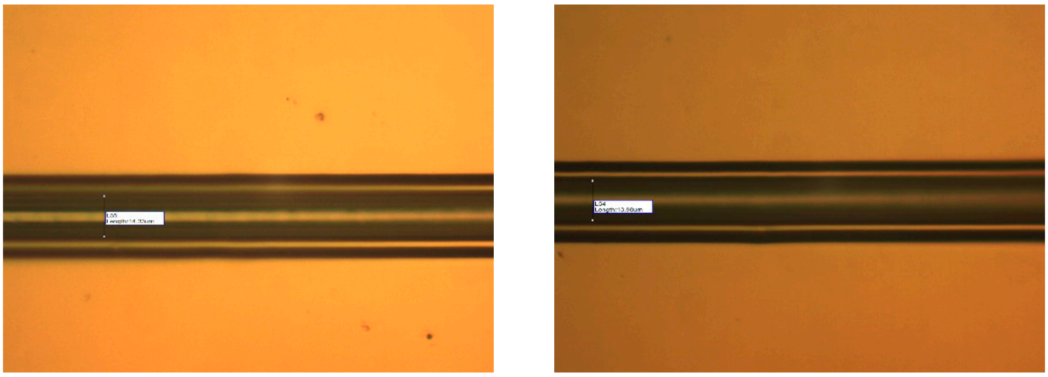

11mW

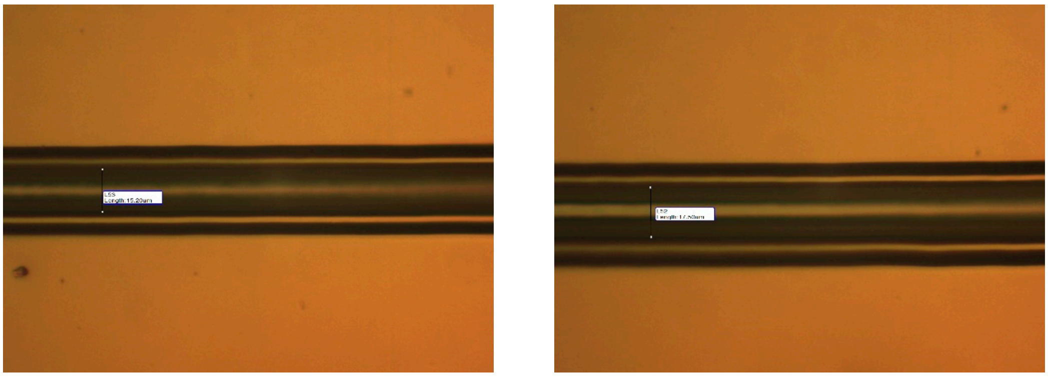

12mW

Channel Width S

Channel width S as a function of the focal plane distance to the ablated surface Z. The surface is observed by microscope 40x magnification and optical width measurements are taken for different values of Z.

Table of Channel Width[row Z: (column measure 1=S1, column measure 2=S2)].

	S1	S2
Z=-1000	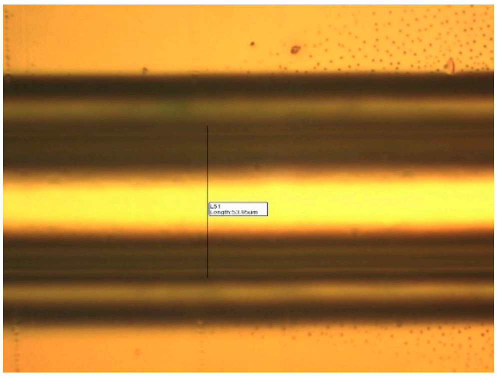	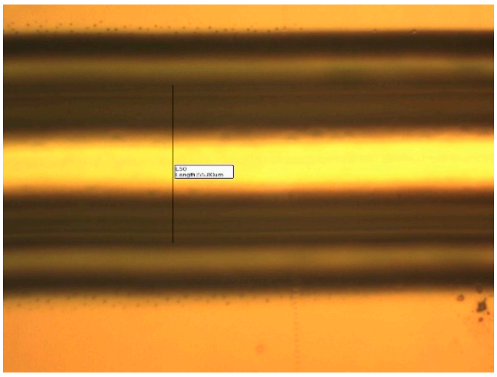
Z=-900	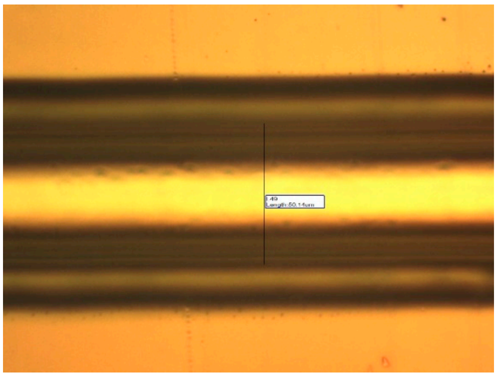	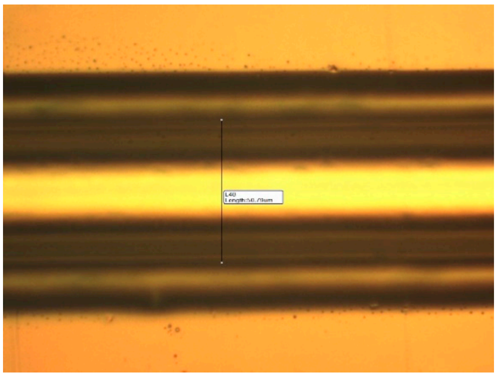
Z=-800	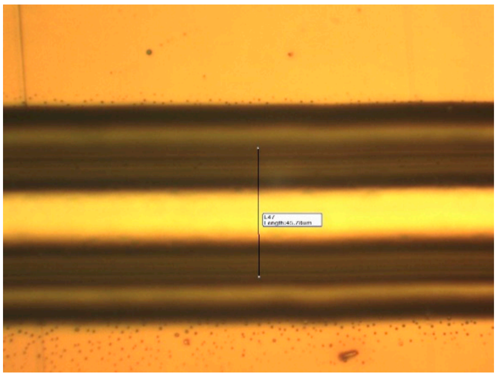	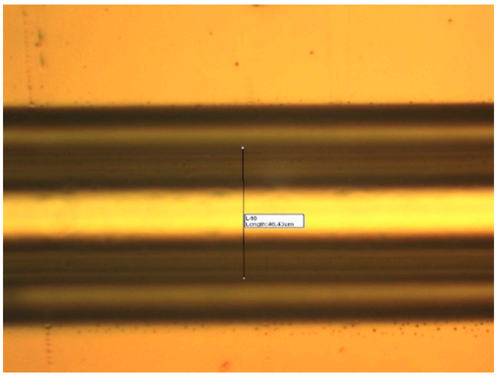
Z=-700	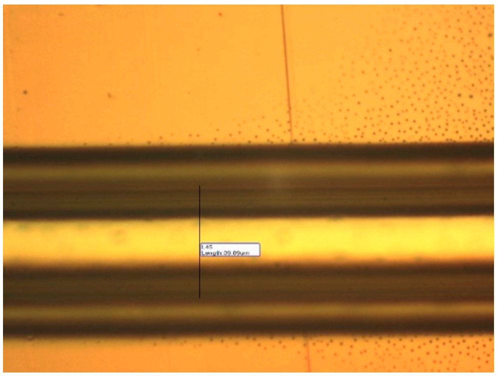	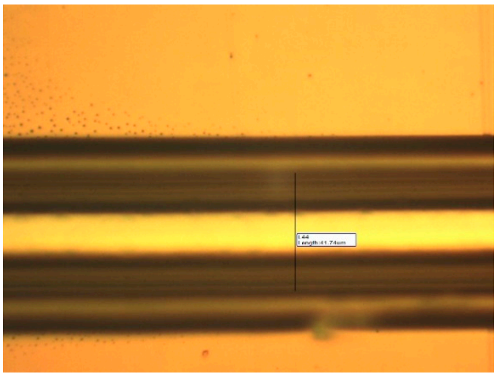

Z=-600

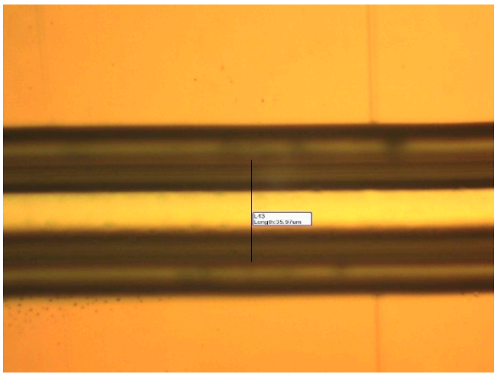

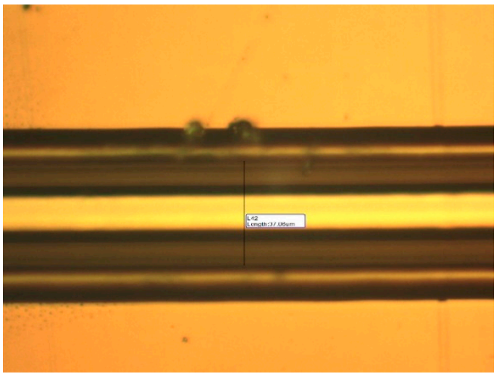

Z=-500

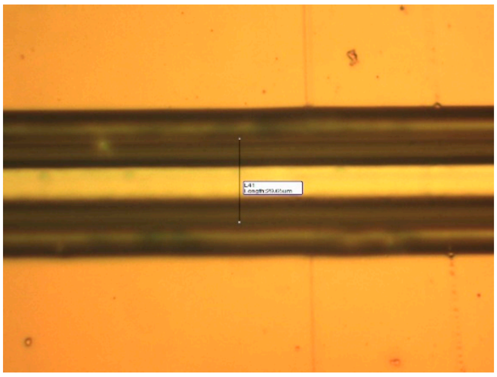

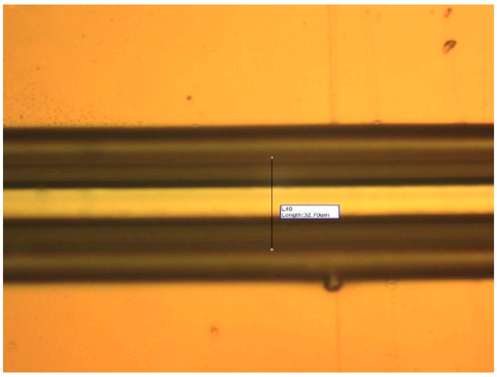

Z=-400

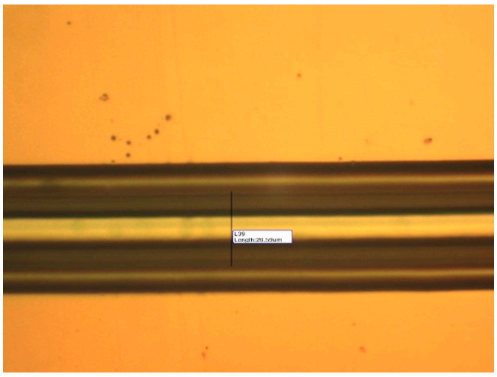

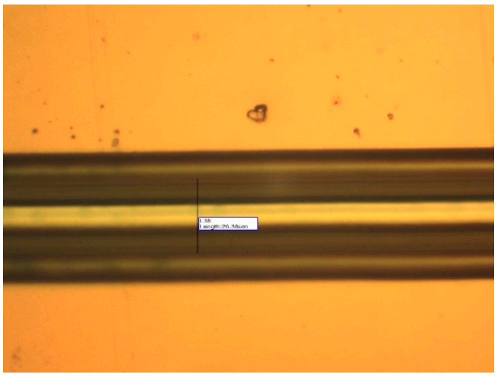

Z=-300

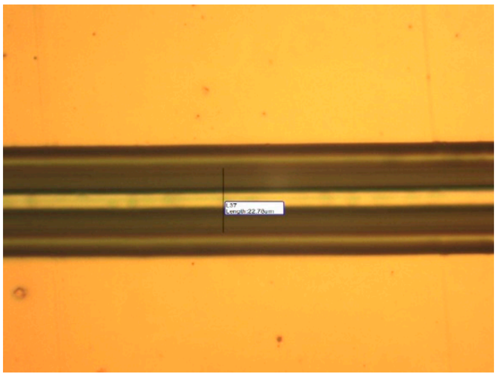

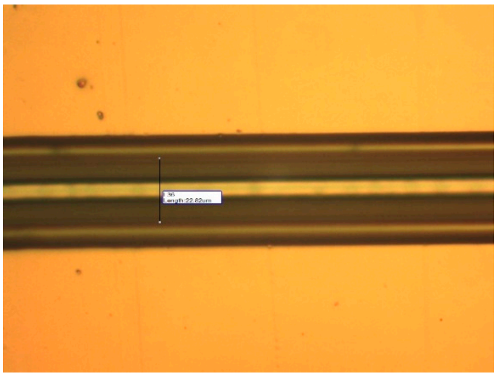

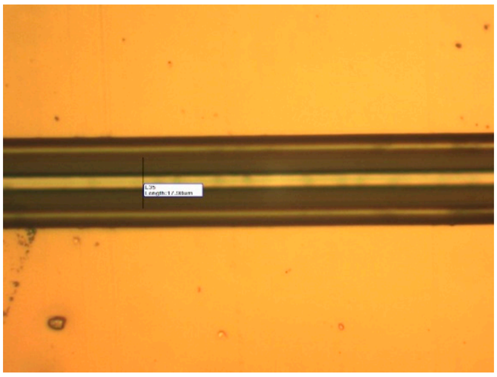
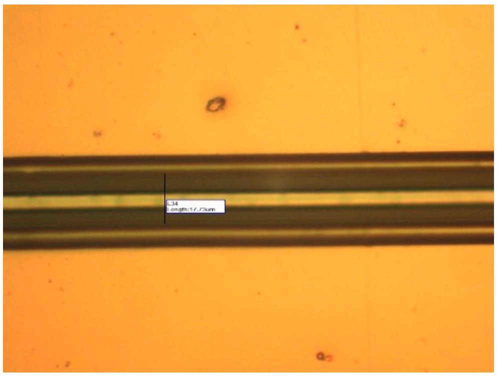

Z=-200

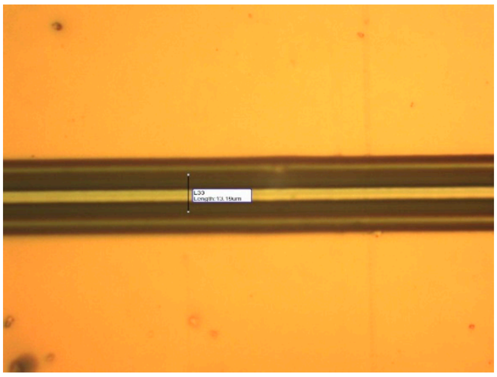
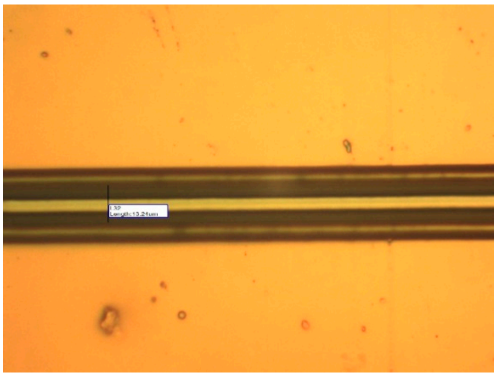

Z=-100

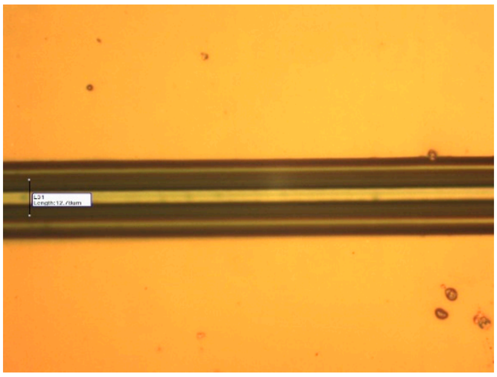
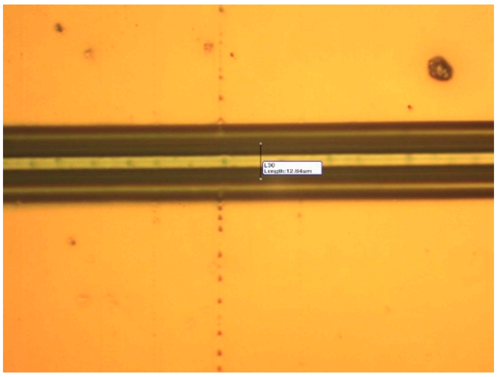

Z=0

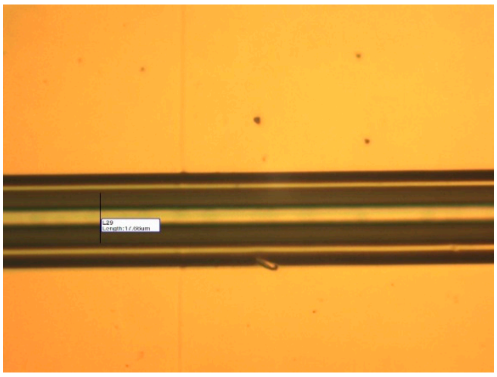
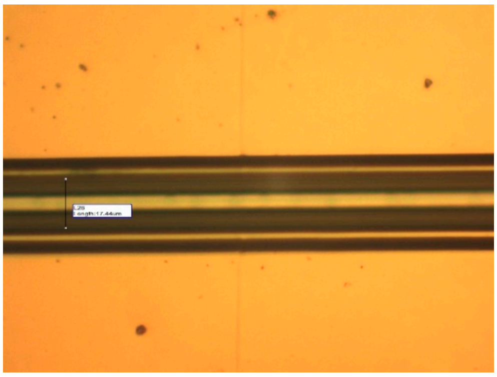

Z=100

Z=200

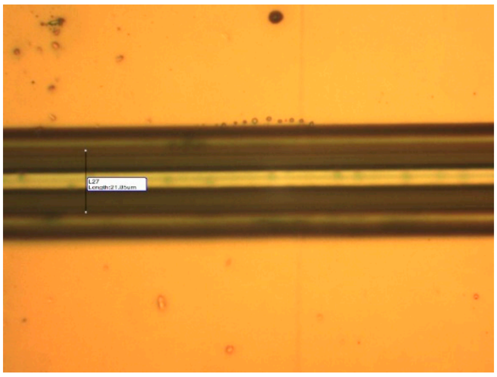
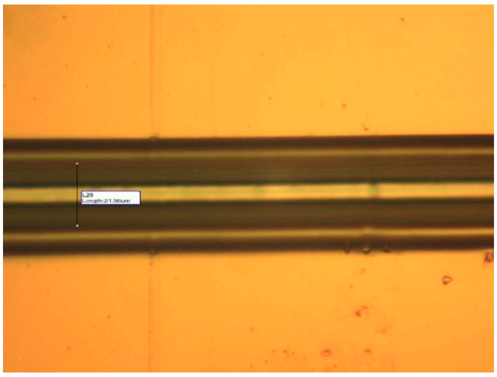

Z=300

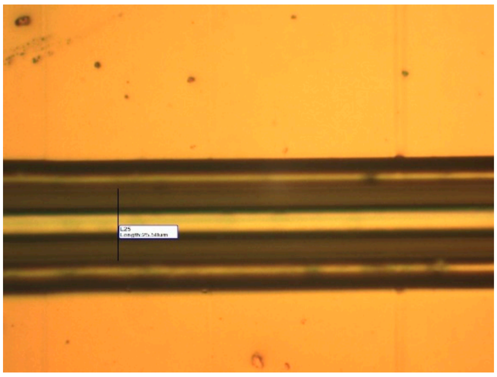
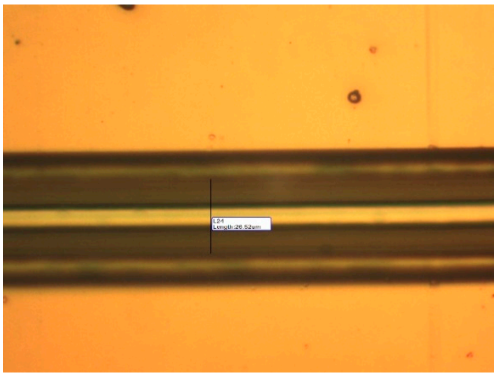

Z=400

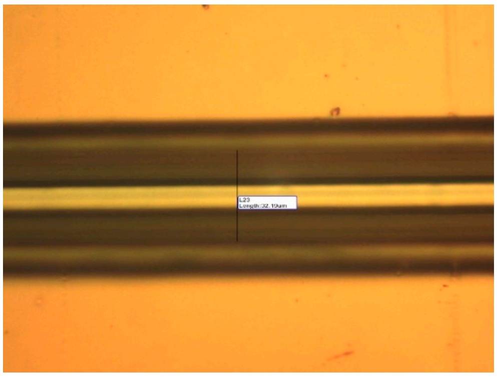
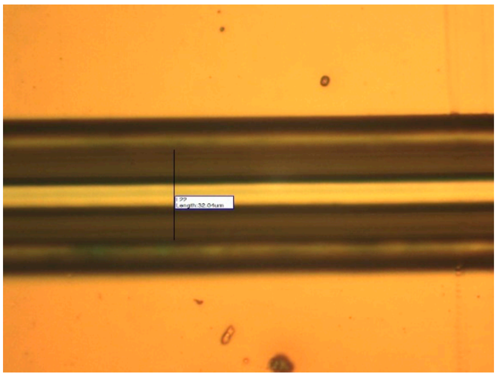

Z=500

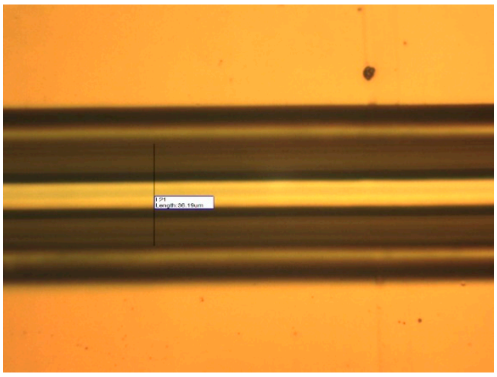
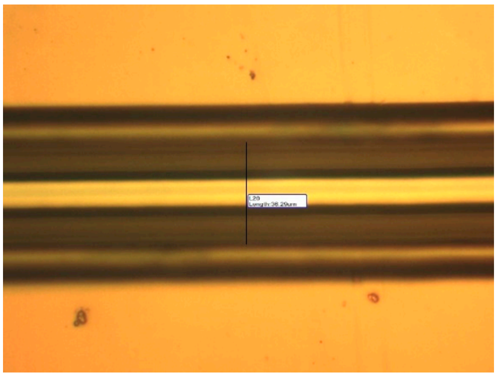

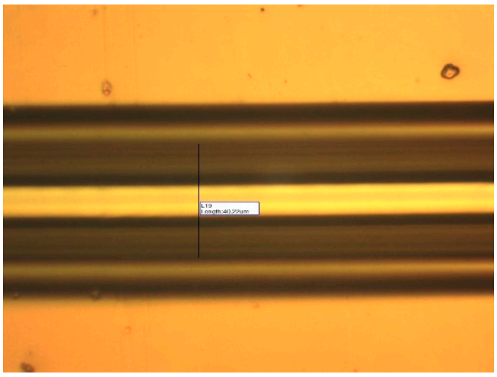
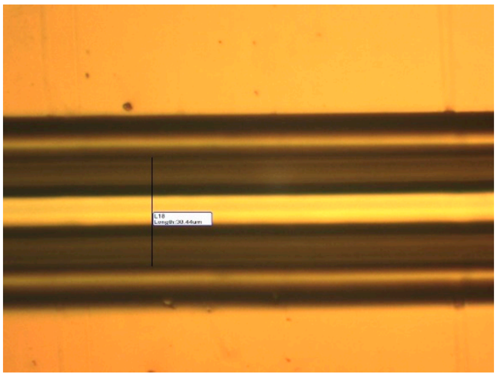

Z=600

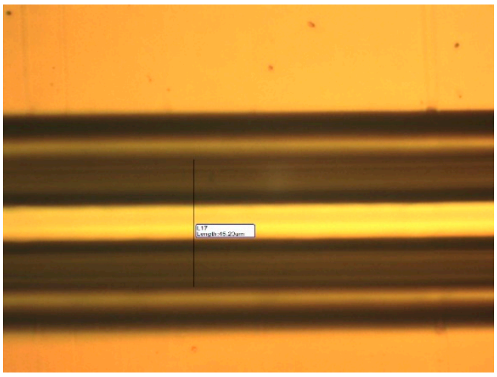
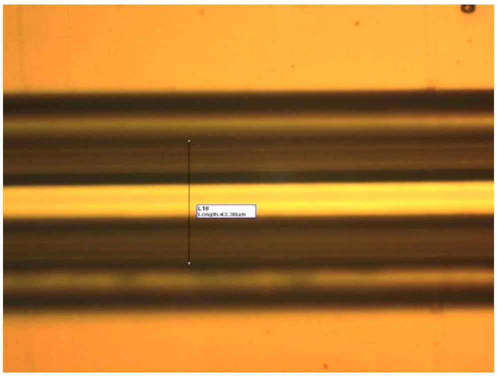

Z=700

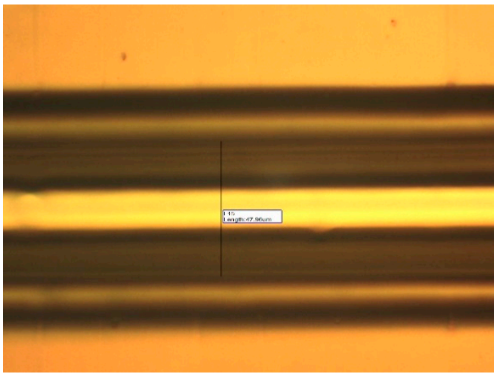
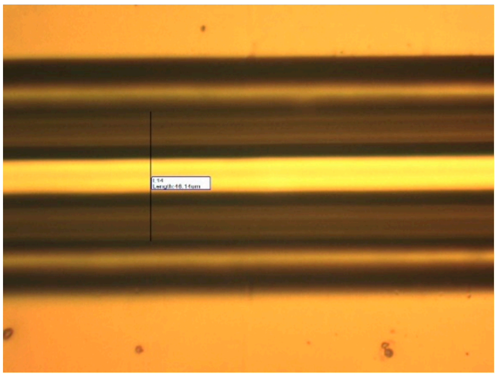

Z=800

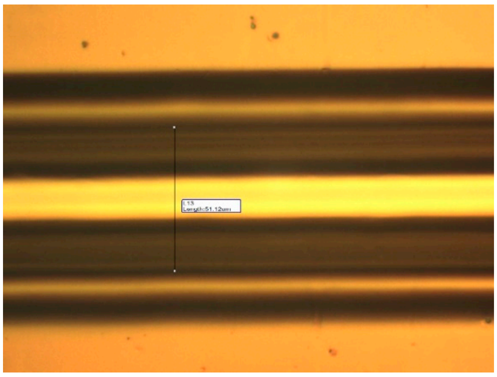
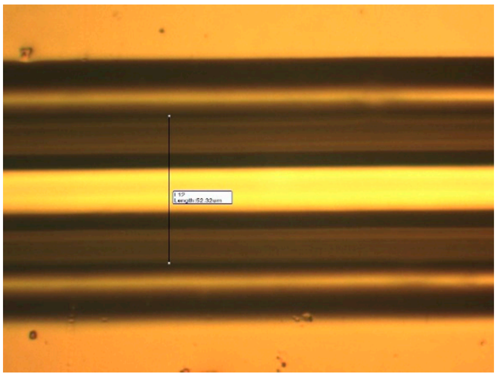

Z=900

Z=1000

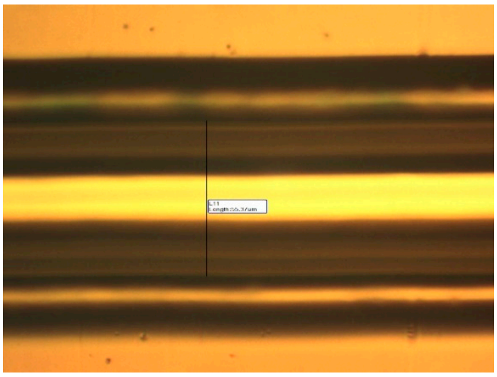

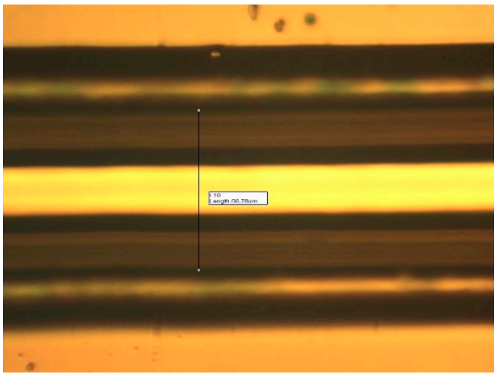

Z=1100

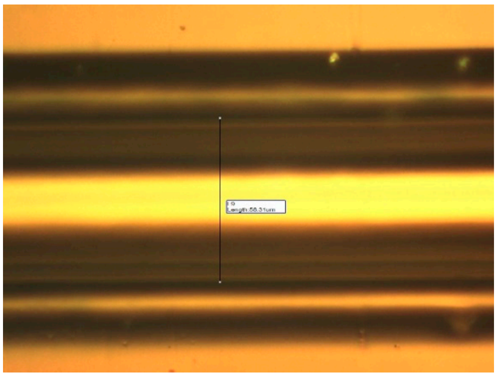

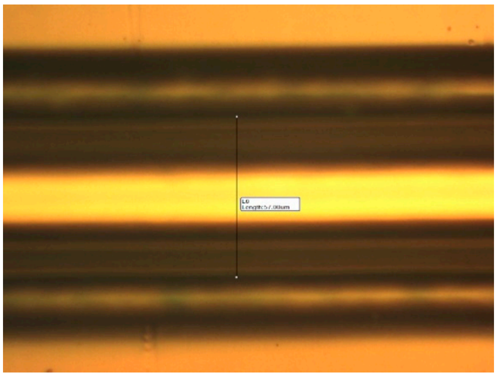

Laser micromachining channel depth.

After exposures, the surface is observed by microscope 40x magnification and optical depth measurements are taken by focusing the upper and lower surfaces of the channel.

3.A) 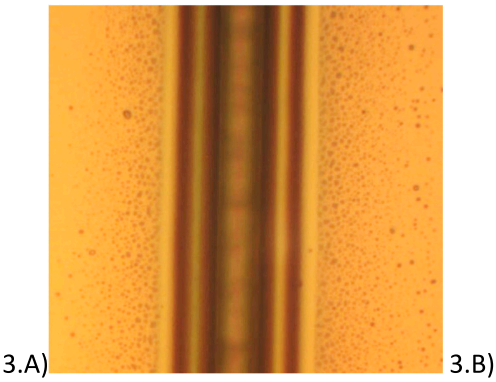 3.B) 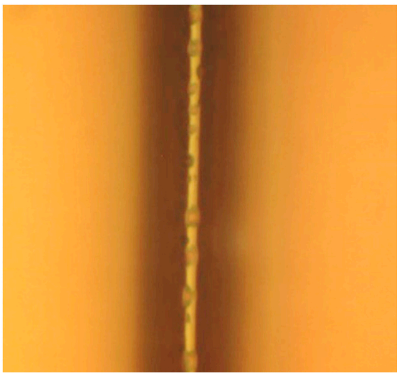

3) The upper (A) and lower (B) surfaces of the channel.
